# Assessment of a Machine Learning Model Applied to Harmonized Electronic Health Record Data for the Prediction of Incident Atrial Fibrillation

**DOI:** 10.1001/jamanetworkopen.2019.19396

**Published:** 2020-01-17

**Authors:** Premanand Tiwari, Kathryn L. Colborn, Derek E. Smith, Fuyong Xing, Debashis Ghosh, Michael A. Rosenberg

**Affiliations:** 1 Colorado Center for Personalized Medicine, University of Colorado School of Medicine, Aurora; 2 Colorado School of Public Health, Department of Biostatics and Informatics, University of Colorado Denver, Aurora; 3 Children’s Hospital Colorado, Cancer Center Biostatistics Core, Department of Pediatrics, University of Colorado, Aurora; 4 Division of Cardiology and Cardiac Electrophysiology, University of Colorado School of Medicine, Aurora

## Abstract

**Importance:**

Atrial fibrillation (AF) is the most common sustained cardiac arrhythmia, and its early detection could lead to significant improvements in outcomes through the appropriate prescription of anticoagulation medication. Although a variety of methods exist for screening for AF, a targeted approach, which requires an efficient method for identifying patients at risk, would be preferred.

**Objective:**

To examine machine learning approaches applied to electronic health record data that have been harmonized to the Observational Medical Outcomes Partnership Common Data Model for identifying risk of AF.

**Design, Setting, and Participants:**

This diagnostic study used data from 2 252 219 individuals cared for in the UCHealth hospital system, which comprises 3 large hospitals in Colorado, from January 1, 2011, to October 1, 2018. Initial analysis was performed in December 2018; follow-up analysis was performed in July 2019.

**Exposures:**

All Observational Medical Outcomes Partnership Common Data Model–harmonized electronic health record features, including diagnoses, procedures, medications, age, and sex.

**Main Outcomes and Measures:**

Classification of incident AF in designated 6-month intervals, adjudicated retrospectively, based on area under the receiver operating characteristic curve and F1 statistic.

**Results:**

Of 2 252 219 individuals (1 225 533 [54.4%] women; mean [SD] age, 42.9 [22.3] years), 28 036 (1.2%) developed incident AF during a designated 6-month interval. The machine learning model that used the 200 most common electronic health record features, including age and sex, and random oversampling with a single-layer, fully connected neural network provided the optimal prediction of 6-month incident AF, with an area under the receiver operating characteristic curve of 0.800 and an F1 score of 0.110. This model performed only slightly better than a more basic logistic regression model composed of known clinical risk factors for AF, which had an area under the receiver operating characteristic curve of 0.794 and an F1 score of 0.079.

**Conclusions and Relevance:**

Machine learning approaches to electronic health record data offer a promising method for improving risk prediction for incident AF, but more work is needed to show improvement beyond standard risk factors.

## Key Points

**Question:**

Can machine learning approaches applied to harmonized electronic health record data identify patients at risk of 6-month incident atrial fibrillation with greater accuracy than standard risk factors?

**Findings:**

This diagnostic study used electronic health record data from more than 2 million individuals to classify patients diagnosed with incident atrial fibrillation within a 6-month period, comparing several approaches to data management. A strategy that included the use of the 200 most common electronic health record features, random oversampling, and a single-layer neural network provided optimal classification of 6-month incident atrial fibrillation; however, this model was only marginally better than a logistic regression model with age, sex, and known risk factors for atrial fibrillation.

**Meaning:**

Machine learning approaches applied to electronic health record data hold promise for predicting clinical outcomes, such as incident atrial fibrillation, but this model was not substantially more accurate than a logistic regression model with standard risk factors.

## Teaser

This diagnostic study examines machine learning approaches applied to harmonized electronic health record data for identifying risk of atrial fibrillation.

## Introduction

Atrial fibrillation (AF) is the most common sustained cardiac arrhythmia, and its prevalence is increasing^1^; approximately 5.1 million individuals had AF in 2010, and an increase to between 9.3 and 12.1 million is anticipated by 2030.^2^ Importantly, the increased risk of mortality with AF is almost entirely because of an increased risk of thromboembolic stroke.^3,4^ This risk could be reduced if a patient with AF and moderate or high risk used oral anticoagulation medication.^5,6,7,8,9,10,11,12,13,14,15,16,17^ A major challenge in the management of patients with AF is that stroke is often the first presentation of AF,^18^ indicating that simply waiting for a patient to develop AF may not be the optimal approach to limiting the risk of stroke. On the other hand, population-wide screening for AF is not currently recommended,^19,20,21^ although some suggest that targeted screening may be useful.^21^ A model that could predict risk of 6-month incident AF could be applied to target screening and identify a patient with AF before their next clinic visit.

The promise of electronic health record (EHR) data has included the potential to leverage so-called big data analytic approaches to predict clinical outcomes in a real-world context. However, despite widespread adoption of EHRs as mandated under the Patient Protection and Affordable Care Act,^22^ there are limited examples of practical applications of EHR data to predict a meaningful clinical outcome.^23,24,25,26^ In addition to the technical limitations of working with data at the scale of the EHR, there are also challenges in performing external validation across health care systems.^27,28,29^ Nonetheless, with the increasing availability of cloud computing platforms^24^ and data storage^30,31^ as well as scalable computational models that can be developed and potentially shared across health care systems, opportunities to apply EHR data to clinical decision-making are emerging. For identifying patients at risk of AF, if an automated prediction algorithm could be applied to patient EHR data, then a clinical decision support system could be developed to guide clinicians toward aggressive screening approaches using standard wearable^32^ or implantable telemetry^33^ devices.

A great deal of enthusiasm has accompanied the potential application of deep learning^34^ and artificial intelligence to outperform humans in image recognition,^35,36^ text recognition,^37,38^ and games,^39^ including checkers^40^ and Go.^41^ However, within the health care setting, the so-called black box characteristic of machine learning (ML) has caused hesitancy in application. In certain situations, ML approaches, such as support vector machines^42^ or random forests,^43^ have been found to produce greater predictive performance than standard regression models.^44,45,46^ More recently, there has been an increased recognition that deep learning models,^34,47^ which are composed of multiple hidden layers of a neural network rather than a single layer, are better equipped to handle the large amount of data that exists in EHRs. However, to understand how these approaches can be applied to a clinical situation, such as the prediction of incident AF, additional study is needed.

In this investigation, we developed and tested an ML model to predict 6-month incidence of AF using EHR data. This time horizon provided a clinically relevant period of prediction, during which patients could undergo screening tests with wearable monitors or electrocardiograms before a follow-up visit. We conducted a systematic examination of EHR data sampled from 2.2 million individuals, in which we harmonized 26 000 features, including diagnoses and medications, under the Observational Medical Outcomes Partnership Common Data Model (OMOP-CDM). During this developmental process, we also identified the appropriate data resampling method to manage data set imbalance and developed a classification algorithm based on training time and accuracy.

## Methods

The code used for the analyses as well as the model weights and mapping (ie, OMOP-CDM input codes) for the final model are available in the eAppendix in the Supplement. The study protocol was approved for analysis of deidentified data (ie, limited data set with dates included) by the University of Colorado institutional review board. Informed consent was waived for practical reasons because of the sample size and deidentification of data. This report adheres to the Transparent Reporting of a Multivariable Prediction Model for Individual Prognosis or Diagnosis (TRIPOD) reporting guideline.^48^

### Study Population and Case Ascertainment

The UCHealth hospital system includes 3 large regional centers (North, Central, and South) in the front range of Colorado that share a single EHR, which allows data from all centers to be pooled into a single data warehouse, a copy of which is located on a cloud platform. This warehouse of data was queried using BigQuery (Google) to create a data set and conduct analyses directly on the cloud platform, where an array of ML tools can be run on virtual machines. To create our study data set, we applied a classification approach based on predicting risk of incident AF during a 6-month period. We performed a structured query language query on the UCHealth EHR for patients with a new diagnosis of AF obtained during a 6-month period. To identify cases, we filtered out all patients with prevalent AF on their first encounter and then at 6-month intervals from each encounter. Patients were assigned a case classification if they had AF diagnosed by *International Classification of Diseases, Ninth Revision *(*ICD-9*) code 427.31 or *ICD*-*10* code I48.91 within that interval. Once a patient was designated a case, they were removed from the pool, and all patients without AF were designated as controls. Data were available in the EHR from January 1, 2011, to October 1, 2018, for 2 252 219 patients.

### CDM and Data Splitting

To offer others the opportunity to validate the findings of this study, we used a CDM for EHR data based on the Observational Health Data Sciences and Informatics collaboration, which uses the OMOP-CDM.^49^ The OMOP-CDM maps raw EHR data to a harmonized data set; for this investigation, we used this CDM with 26 000 variables (ie, features) from the EHR, including age, sex, diagnoses, procedures, and medications. Additional data, including laboratory values and relevant cardiovascular studies (eg, electrocardiograms), have not been mapped to OMOP-CDM in our system and were excluded from this investigation. These values were time-stamped with the time of entry into the medical record, which was used to associate the value with the timing of the outcome of interest. Features were encoded using 1-hot encoding, which assigned a value of 1 if that feature was present for that individual and 0 otherwise, and were collected cumulatively from the time of first encounter until diagnosis of AF (for cases) or until end of follow-up (for controls). Preliminary studies identified a substantial decrease in analytical time using the 200 most common concepts among the complete EHR. These were used as input into prediction models (eTable 1 and eTable 2 in the Supplement). The final data set was composed of 2 252 219 million records, which were then split into training (1 801 775 records [80.0%]) and testing (450 444 records [20.0%]) sets to compare the models developed in this investigation.

### Model Development

For all models, hyperparameter tuning to identify the optimal values for parameters that are not learned during the training process (ie, learning rate) was performed using iterative random sampling of 10 000 records for manual grid search (for neural networks) and 10-fold cross-validation for automated grid search (for other ML approaches). Further details appear in the eMethods in the Supplement.

Because of the relative infrequency of the outcome (ie, 6-month incident AF) across the data set, there was an imbalance between the cases and controls. In the presence of imbalance, many classification algorithms, which often base classification on a probability of disease of greater than 50%, will take longer or even fail to achieve convergence. Approaches to rebalance data, which match the number of cases to the number of controls, include oversampling (ie, adding cases to match the number of controls), undersampling (ie, reducing the number of controls to match the number of cases), and hybrid approaches. We examined several strategies for resampling, including random oversampling, the synthetic minority oversampling technique,^50^ random undersampling, and cluster centroid undersampling. To identify the best resampling approach, we used a deep neural network (7 layers with 100 neurons/layer) because pilot analyses using a smaller data set suggested this approach might be superior to other ML approaches. Each resampling approach was applied to the training data set and then compared using the testing set. We also compared the resampled training set with a model using no resampling (ie, an imbalanced set).

Once we identified an optimal resampling approach, we compared several classification algorithms, including naive Bayesian classification, regularized logistic regression, random forest classification, gradient boosted classification, 1-layer fully connected neural networks (shallow), and multiple-layer fully connected neural networks (deep). Model comparison was based on area under the receiver operating characteristic curve and the F1 statistic.^51,52,53^ Naive Bayesian classification algorithms^54^ apply Bayes theorem to predict the probability of disease classification based on the assumption of independence (ie, naive) between predictors. Regularized logistic regression (ie, ridge regression) applies a shrinkage penalty to predictors to avoid overfitting. Random forest classification^55^ algorithms create a multitude of decision trees using different subsets of the predictors, from which the appropriate class is selected as the mode of the collection of trees. Gradient boosted classification^56^ algorithms are similar to random forests, except they use a stepwise process to reduce the misclassification error of prior models. Neural networks,^34,47,57^ which can be shallow (ie, 1 layer) or deep (ie, multiple layers), are interconnected nodes, each of which is essentially a nonlinear regression equation, all of which are combined to minimize a loss function; in this study, the loss applied is the cross-entropy loss. Computation time includes all prior data sampling and algorithm performance. Once an optimal model and resampling approach were identified, we conducted sensitivity analyses using several alternative resampling and modeling approaches in combination to ensure that the combination (ie, dimensionality reduction, resampling, and classification algorithm) identified was indeed optimal. Precision recall and receiver operating characteristic curves as well as feature importance plots were created for the optimal model for manual inspection.

### Validation of Developed Model

The optimal model was compared with an unregularized logistic regression model based on the presence of known clinical predictors of AF. These included age, sex, hypertension (*ICD-9 *code, 401.x; *ICD-10 *code, I10), obesity (*ICD-9 *code, 278.0; *ICD-10 *code, E66.9), diabetes (*ICD-9 *code, 250.0; *ICD-10 *code, E11.9), coronary artery disease (*ICD-9 *code, 414.01; *ICD-10 *code, I25.1x), mitral valve disease (*ICD*-*9 *code, 394.0 or 424.0; *ICD*-10 code, I34.2 or I34.0), heart failure (*ICD-9 *code, 428.0; *ICD-10 *code, I50.9), and chronic kidney disease (*ICD*-*9 *code, 585.9; *ICD*-*10 *code, N18.9).

### Statistical Analysis

Model comparison was performed using the area under the receiver operating characteristic curve for classification accuracy of 6-month incident AF and the *F1 score*, defined as the harmonic mean of precision (ie, positive predictive value) and recall (ie, sensitivity), with perfect precision and recall at an F1 score of 1 and the worst precision and recall at 0. All analyses were run on the Google Cloud Platform (Google), using 96 central processing units and 620 gigabytes of random access memory. Scripts were composed in Python version 3 (Python) and were run on Jupyter Notebook (Project Jupyter) with Tensorflow platform (Google) on the Google Cloud Platform. Machine learning packages included scikit-learn and keras*.* Confidence intervals were calculated using the Wald method,^58,59^ although almost all were within the rounding error of the estimates because of the large testing sample size (ie, approximately 440 000) and are not displayed. We did not perform formal null-hypothesis testing, so no prespecified level for statistical significance was set. Additional details appear in the eMethods in the Supplement.

## Results

Across the UCHealth population of 2 252 219 individuals, with 1 225 533 women (54.4%) and a mean (SD) age of 42.9 (22.3) years, we identified 28 036 patients (1.2%) with 6-month incident AF (Table 1). Cardiac disease and known risk factors were more common among patients who developed incident AF than among those with no AF, including older age (mean [SD] age, 71.7 [16.5] years vs 42.9 [22.3] years) and male sex (12 919 [46.1%] women vs 1 212 586 [54.5%] women) (Table 1).

**Table 1.** UCHealth Population by AF Diagnosis

Characteristic^a^	Patients, No. (%)	
	No AF (n = 2 224 183)	6-mo Incident AF (n = 28 036)
Age, mean (SD), y	42.86 (22.26)	71.65 (16.47)
Women	1 212 586 (54.51)	12 919 (46.08)
Hypertension	358 347 (16.11)	13 349 (47.60)
Coronary artery disease	64 183 (2.88)	3830 (13.66)
Mitral valve disease	23 192 (1.04)	1974 (7.04)
Heart failure	34 806 (1.56)	2906 (10.36)
Diabetes	126 941 (5.7)	4780 (17.04)
Obesity	123 564 (5.55)	2715 (9.68)
Chronic kidney disease	38 834 (1.74)	2229 (7.95)

Abbreviation: AF, atrial fibrillation.

^a^ Diagnoses based on presence of *International Classification of Diseases, Ninth Revision* (*ICD*-*9*) or *ICD*-*10* codes, as follows: hypertension, *ICD*-*9 *code, 401.x; *ICD*-*10 *code, I10; coronary artery disease, *ICD*-*9 *code, 414.01; *ICD*-*10 *code, I25.1; mitral valve disease, *ICD*-*9 *code, 394.0 or 424.0; *ICD*-*10 *code, I34.2 or I34.0; heart failure, *ICD*-*9 *code, 428.0; *ICD*-*10 *code, I50.9; diabetes, *ICD*-*9 *code, 250.0; *ICD*-*10 *code, E11.9; obesity, *ICD*-*9 *code, 278.0; *ICD*-*10 *code, E66.9; and chronic kidney disease, *ICD*-*9 *code, 585.9; *ICD*-*10 *code, N18.9.

We first examined the role of undersampling and oversampling methods to identify the optimal approach to manage the imbalance between cases and controls that we identified in this data set. Using a 7-layer deep neural network algorithm with hyperbolic tangent activation and 20% dropout, we found that random oversampling provided the best classification F1 score (0.101) compared with other methods (eg, synthetic minority oversampling technique, 0.090; random undersampling, 0.099), including no resampling, which provided substantially poorer classification (0.002) than other methods (Table 2).

**Table 2.** Comparison of Resampling Strategies^a^

Strategy	F1 Score	AUC	Training Time, min
Oversampling			
Random	0.101	0.800	17.1
Synthetic minority oversampling technique	0.090	0.786	22.3
Undersampling			
Random	0.099	0.808	5.4
Cluster centroid	0.062	0.743	50.8
None	0.002	0.500	10.2

Abbreviation: AUC, area under the receiver operating characteristic curve.

^a^ Sampling comparison from deep learning model.

Using the random oversampling strategy, we examined several classification algorithms to identify a potential overall best model. Among the approaches examined, we found that a single-layer shallow neural network using the 200 most common EHR features, including age and sex, was superior to other methods (F1 score, 0.110; AUC, 0.800), which included regularized regression (F1, 0.088; AUC, 0.806), gradient boosted descent (F1, 0.108; AUC, 0.762), random forest classification (F1, 0.076; AUC, 0.792), and a deep neural network (F1, 0.101; AUC, 0.800) (Table 3). The single-layer neural network had a specificity of 84.9%, a sensitivity (ie, recall) of 75.2%, negative predictive value of 99.6%, and positive predictive value of 5.9% at a probability (ie, decision) cutoff of 0.5, with relatively poor calibration across predicted probabilities (Figure; eTable 3A and eFigure in the Supplement).

**Table 3.** Comparison of Machine Learning Approaches^a^

Approach	F1 Score	AUC	Training Time, min
Naive Bayes	0.059	0.647	1.2
Logistic regression with L2 regularization	0.088	0.806	66.2
Random forest	0.076	0.792	3826.8
Neural network			
Shallow	0.110	0.800	666.1
Deep	0.101	0.800	17.1
Gradient boosted machine	0.108	0.762	17 223.4

Abbreviation: AUC, area under the receiver operating characteristic curve.

^a^ Using random oversampling and all features. F1 score and AUC were calculated from model applied to held-out testing set (20%); training time was for training of training set (80%).

**Figure.**
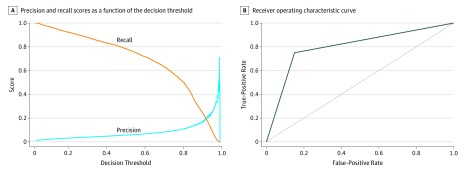
Precision Recall and Area Under the Receiver Operating Characteristic Curve for the Optimal Model A, Because of the low incidence of atrial fibrillation systemwide, most decision thresholds did not have a high recall (positive predictive value). See text for details. B, Area under the receiver operating characteristic curve was 0.80.

Finally, we compared the optimal model with an unregularized logistic regression model based on known AF risk factors. The logistic regression model had an F1 score of 0.079 and AUC of 0.794 (eTable 3B and eTable 4 in the Supplement).

## Discussion

In this investigation of the application of an ML model using harmonized EHR data for identifying patients at risk of 6-month incident AF, we found that a shallow neural network using random oversampling provided the optimal classification of risk, although this model was not substantially better than a simpler model based on known AF risk factors. These results are important because, in addition to motivating future investigations to apply ML methods to EHR data to identify patients at risk of AF, they also incorporated harmonized data. This means that the optimal model can be directly applied not only in our institution’s EHR but in data from the EHR of any other medical institution participating in OMOP or the Observational Health Data Sciences and Informatics collaboration. In clinical application, our model could thus be inserted directly in the user interface to guide targeted screening of patients at risk of AF, including the development of prospective follow-up studies to use the prediction for targeted screening for AF, such as routine electrocardiograms, implantable devices, or wearable devices.

However, there are several reasons for hesitancy before taking these results directly to the bedside to guide clinical management without additional investigation. First, the model that we identified was not extremely accurate, with an F1 score under 20% and a sensitivity of approximately 75% based on a cutoff probability of 0.5 for risk. Although the probability threshold could be lowered to improve sensitivity of classification, the decreased specificity and number of false-positive results with such an approach would result in a large number of patients undergoing unfruitful screening. Second, as mentioned, we found that a simple logistic regression model based on known and clinical risk factors^60,61,62,63^ performed nearly as well as the more sophisticated ML model. This limitation has practical importance because many EHR systems allow for built-in regression modeling that can be plugged directly into the system in real time.

In the process of developing a 6-month risk prediction model for AF, we made several important observations about the application of ML to EHR data. First, we found that, for a rare condition like 6-month incident AF (which occurred in 1.2% of our sample), oversampling to rebalance the data was superior to using the imbalanced data set. The limitations of applying classification algorithms to imbalanced data sets are well described,^64,65,66^ and yet many investigations continue to use imbalanced data to draw conclusions from statistical modeling.

Second, we found that a shallow neural network provided the optimal classification algorithm, with better classification than a deep neural network approach. This finding demonstrates that, although deep learning approaches may be superior for classification of structured data sets such as in image^67^ or voice^68^ recognition, they do not always perform better than other standard ML algorithms. This finding also highlights the importance of examining all approaches for each classification problem, rather than assuming a given approach is optimal.

There are several additional strengths of this investigation. First, all models were created using a harmonization scheme (ie, OMOP-CDM) that could allow for direct application and validation to data mapped from a separate EHR. Such harmonization allows for exploring transfer-learning^69^ approaches, which could provide additional insights into similar and divergent AF risk factors across populations. Second, we conducted a systematic approach to identify the best resampling and classification algorithm for this outcome. Further work in other outcomes is needed to determine if the combination we identified for predicting 6-month incident AF would also be optimal for prevalent or longer-term AF predictions as well as for outcomes that are more or less common than AF. Finally, we examined a data set with more than 2 million individuals, which provided a large enough sample size from our single institution to conduct cross-validation and out-of-sample validation. This power from the use of big data is possible because of the unique circumstances of our relationship with the Google Cloud Platform. However, many other EHRs are moving to the cloud, providing further opportunities for development and testing.

### Limitations

There are several limitations in this study, many of which are the subject of future, more targeted investigations. First, our study included a very simple method for managing the temporal relationships between features in our data set, which did not account for time-varying effects or censoring. An AF event that occurred the day after an encounter was modeled the same as an AF event occurring the day before a subsequent encounter, and a diagnosis or medication that was given a month before the AF diagnosis was weighted the same as that given 4 years prior. While we suggest that the approach we used for this investigation was reasonable based on the typical 6-month follow-up schedule for patients seen in cardiology clinics, we realize that additional information about temporal risk will be needed for more accurate prediction approaches. More sophisticated methods such as recurrent neural networks^70,71^ or parametric survival functions^72^ could provide more accurate predictions in future investigations. A second limitation is that we excluded some data elements, such as laboratory values and diagnostic test results, that may have had prognostic value for predicting 6-month AF.^73,74^ Some of these values have been difficult to harmonize across data sets via OMOP-CDM, and others, such as echo measures of diastolic function, have high variability in interinstitutional measurement.^62,75^ Nonetheless, there are many additional biomarkers^73,74^ likely to have a more biological association with risk of AF than a diagnostic association, and future applications that include this information would be expected to provide both predictive and inferential knowledge about the risk of AF. Third, despite having a reasonably good AUC for the classification of 6-month risk of AF (ie, 0.80), the actual increase in risk based on the model is not particularly elevated (5.9% for positive predicted risk, and 0.4% for negative prediction) because of the overall low incidence of AF across the sample. Clinically, we believe that this limitation indicates that our model approach would be insufficient for guiding treatments, such as initiating anticoagulation medication, but we suggest that that it could be used to identify individuals who may or may not be candidates for enhanced screening, eg, through the use of wearable monitors. Further prospective studies would be needed to identify the actual benefit that could be obtained from integration of this approach into a screening program for AF. However, we believe that because our model was constructed directly from EHR data, integration into a systemwide screening program would be more practical than if the model were created using data that needed to be collected outside the EHR. Also, although the systematic harmonized approach we used in this study holds potential for cross-institutional validation, much work is needed in terms of data sharing before actual testing can be performed. We did not have data on race/ethnicity or socioeconomic status readily available in our data set, but we are certain that our population is likely to be different from those cared for in many other health systems, and we believe that it is only through external validation in other EHRs that an unbiased risk prediction model could be developed. Our group and others are working in this direction, and the hope is that, sometime soon, all EHRs will incorporate a standard risk prediction model for AF and many other conditions.

## Conclusions

We studied the development of an ML model for predicting 6-month risk of AF using harmonized EHR data and found that the combination of random oversampling and single-layer neural network classification provided superior prediction than other ML models. Further work is needed to explore the technical and clinical applications of this model to improving outcomes.

## Notes

Supplement. **eAppendix.** Script of Python Code Used for Analysis **eMethods.** Machine Learning Code Details **eTable 1.** Top 30 Features Across Entire Population **eTable 2.** List of OMOP Concept Numbers for Features Used as Input **eTable 3.** Confusion Matrices **eTable 4.** Coefficients for Logistic Regression Model **eFigure.** Calibration Curve of Final Model
