# Supplementary material for: Assessment of a Machine Learning Model Applied to Harmonized Electronic Health Record Data for the Prediction of Incident Atrial Fibrillation
Source: JAMA Netw Open. 2020 Jan 17;3(1):e1919396. doi: 10.1001/jamanetworkopen.2019.19396 (PMC6991266; doi:10.1001/jamanetworkopen.2019.19396)
Supplement: Supplement. — eAppendix. Script of Python Code Used for Analysis eMethods. Machine Learning Code Details eTable 1. Top 30 Features Across Entire Population eTable 2. List of OMOP Concept Numbers for Features Used as Input eTable 3. Confusion Matrices eTable 4. Coefficients for Logistic Regression Model eFigure. Calibration Curve of Final Model [file jamanetwopen-3-e1919396-s001.pdf]

Supplementary Online Content

Tiwari P, Colborn KL, Smith DE, Xing F, Ghosh D, Rosenberg MA. Assessment of a machine learning model applied to harmonized electronic health record data for the prediction of incident atrial fibrillation. *JAMA Netw Open*. 2020;3(1):e1919396. doi:10.1001/jamanetworkopen.2019.19396

eAppendix. Script of Python Code Used for Analysis
eMethods. Machine Learning Code Details
eTable 1. Top 30 Features Across Entire Population
eTable 2. List of OMOP Concept Numbers for Features Used as Input
eTable 3. Confusion Matrices
eTable 4. Coefficients for Logistic Regression Model
eFigure. Calibration Curve of Final Model

This supplementary material has been provided by the authors to give readers additional information about their work.

© 2020 Tiwari P et al. *JAMA Network Open.*

eAppendix. Script of Python Code Used for Analysis

In [ ]:

```
import pandas as pd
import glob
import numpy as np
import google.datalab.bigquery as bq
import pandas as pd
import tensorflow as tf
from tensorflow.contrib import lookup
from tensorflow.python.platform import gfile
import numpy
import keras as kr
from keras.preprocessing.text import Tokenizer
from keras.models import Sequential
from keras.layers import Dense, Activation, Dropout
from keras.layers.advanced_activations import LeakyReLU, PReLU
from keras.utils import multi_gpu_model
import itertools
from sklearn.metrics import confusion_matrix
import matplotlib.pyplot as plt
import datetime
import time
```

© 2020 Tiwari P et al. *JAMA Network Open.*

In [ ]:

```
def plot_confusion_matrix(cm, classes,
                          normalize=False,
                          title='Confusion matrix',
                          cmap=plt.cm.Blues):
    """
    This function prints and plots the confusion matrix.
    Normalization can be applied by setting `normalize=True`.
    """
    plt.imshow(cm, interpolation='nearest', cmap=cmap)
    plt.title(title)
    plt.colorbar()
    tick_marks = np.arange(len(classes))
    plt.xticks(tick_marks, classes, rotation=0)
    plt.yticks(tick_marks, classes)

    if normalize:
        cm = cm.astype('float') / cm.sum(axis=1)[:, np.newaxis]
        #print("Normalized confusion matrix")
    else:
        1#print('Confusion matrix, without normalization')

    #print(cm)

    thresh = cm.max() / 2.
    for i, j in itertools.product(range(cm.shape[0]), range(cm.shape[1])):
        plt.text(j, i, cm[i, j],
                 horizontalalignment="center",
                 color="white" if cm[i, j] > thresh else "black")

    plt.tight_layout()
    plt.ylabel('True label')
    plt.xlabel('Predicted label')
```

In [ ]:

```
%%bq query -n requests
SELECT
label,  age, sex, diag_med
 FROM `hdcmlmed.adhocs.v_afib`
```

In [ ]:

```
data = requests.execute(output_options=bq.QueryOutput.dataframe()).result()
from sklearn.utils import shuffle
data = shuffle(data)

from sklearn.preprocessing import StandardScaler
train_size = int(len(data) * .8)

train_icdmed = data['diag_med'][:train_size]
train_label = data['label'][:train_size]
```

© 2020 Tiwari P et al. *JAMA Network Open.*

```
[illegible]

test_icdmed = data['diag_med'][train_size:]
test_label = data['label'][train_size:]

vocab_size = 200
tokenize = kr.preprocessing.text.Tokenizer(num_words=vocab_size,
                                           filters='!"#$%&()*+,-./:;<=>?@[\\]^_`
{|}~\t\n',
                                           lower=True,
                                           split=",",char_level=False)
tokenize.fit_on_texts(train_icdmed)
x_train = tokenize.texts_to_matrix(train_icdmed)
x_test = tokenize.texts_to_matrix(test_icdmed)

train_sex = data['sex'][:train_size]
test_sex = data['sex'][train_size:]
vocab_size = 2
tokenize = kr.preprocessing.text.Tokenizer(num_words=vocab_size,
                                           filters='!"#$%&()*+,-./:;<=>?@[\\]^_`
{|}~\t\n',
                                           lower=True,
                                           split=",",char_level=False)
tokenize.fit_on_texts(train_sex)
x_train_sex = tokenize.texts_to_matrix(train_sex)
x_test_sex = tokenize.texts_to_matrix(test_sex)

x_train=np.hstack((x_train,x_train_sex))
x_test=np.hstack((x_test,x_test_sex))

scaler = StandardScaler()
age=data['age']
age=scaler.fit_transform(age.reshape(-1,1))
age_train=age[:train_size]
age_test=age[train_size:]
x_train=np.hstack((x_train,age_train))
x_test=np.hstack((x_test,age_test))


from sklearn import preprocessing
encoder = preprocessing.LabelBinarizer()
encoder.fit(train_label)
y_train = encoder.transform(train_label)
y_test = encoder.transform(test_label)


from imblearn.over_sampling import RandomOverSampler
from sklearn import preprocessing


print("Before OverSampling, counts of label '1': {}".format(sum(y_train==1)))
print("Before OverSampling, counts of label '0': {} \n".format(sum(y_train==0)))

sm = RandomOverSampler(random_state=42)
```

© 2020 Tiwari P et al. *JAMA Network Open.*

```
X_train_res, y_train_res = sm.fit_sample(x_train, y_train)

t0 = time.time()

from keras.models import Sequential
from keras.layers import Dense, Activation, Dropout
from keras.layers.advanced_activations import LeakyReLU, PReLU
from keras.utils import multi_gpu_model
from keras.applications import Xception
from keras.callbacks import EarlyStopping

t0 = time.time()

earlystop = EarlyStopping(monitor='val_loss', min_delta=0.0001, patience=1, verb
ose=1, mode='auto')
callbacks_list = [earlystop]
model = Sequential()
model.add(Dense(100, input_shape=(x_train.shape[1],)))
model.add(Activation('tanh'))
model.add(Dropout(0.2))
model.add(Dense(100))
model.add(Activation('tanh'))
model.add(Dropout(0.2))
model.add(Dense(100))
model.add(Activation('tanh'))
model.add(Dropout(0.2))
model.add(Dense(100))
model.add(Activation('tanh'))
model.add(Dropout(0.2))
model.add(Dense(100))
model.add(Activation('tanh'))
model.add(Dropout(0.2))
model.add(Dense(100))
model.add(Activation('tanh'))
model.add(Dropout(0.2))
model.add(Dense(100))
model.add(Activation('tanh'))
model.add(Dropout(0.2))
model.add(Dense(2, activation = 'softmax'))


adam = kr.optimizers.Adamax(lr=0.001, beta_1=0.9, beta_2=0.999, epsilon=1e-08, d
ecay=0.0)
model.compile(loss='sparse_categorical_crossentropy',
              optimizer=adam,
              metrics=['accuracy'])

history=model.fit(X_train_res, y_train_res.ravel(),
                    batch_size=64,
                    epochs=50,
                    callbacks=callbacks_list,
                    validation_split=0.1)

y_softmax = model.predict(x_test)
```

© 2020 Tiwari P et al. *JAMA Network Open.*

```python
y_test_1d = []
y_pred_1d = []

for i in range(0, len(y_softmax)):
    probs = y_softmax[i]
    predicted_index = np.argmax(probs)
    y_pred_1d.append(predicted_index)

labels=[0,1]
def plot_confusion_matrix(cm, classes,
                          normalize=False,
                          title='Confusion matrix',
                          cmap=plt.cm.Blues):
    """
    This function prints and plots the confusion matrix.
    Normalization can be applied by setting `normalize=True`.
    """
    if normalize:
        cm = cm.astype('float') / cm.sum(axis=1)[:, np.newaxis]
        print("Normalized confusion matrix")
    else:
        print('Confusion matrix, without normalization')

    print(cm)

    plt.imshow(cm, interpolation='nearest', cmap=cmap)
    plt.title(title)
    plt.colorbar()
    tick_marks = np.arange(len(classes))
    plt.xticks(tick_marks, classes, rotation=45)
    plt.yticks(tick_marks, classes)

    fmt = '.2f' if normalize else 'd'
    thresh = cm.max() / 2.
    for i, j in itertools.product(range(cm.shape[0]), range(cm.shape[1])):
        plt.text(j, i, format(cm[i, j], fmt),
                 horizontalalignment="center",
                 color="white" if cm[i, j] > thresh else "black")

    plt.tight_layout()
    plt.ylabel('True label')
    plt.xlabel('Predicted label')

# Compute confusion matrix
cnf_matrix = confusion_matrix(y_test, y_pred_1d)
np.set_printoptions(precision=2)

from sklearn.metrics import f1_score
print("f-score metric in the testing dataset: {}%".format(f1_score(y_test, y_pre
d_1d, average='binary') ))

from sklearn import metrics
```

© 2020 Tiwari P et al. *JAMA Network Open.*

```
fpr, tpr, thresholds = metrics.roc_curve(y_test, y_pred_1d)

print("AUC of test dataset is: {}" .format(metrics.auc(fpr, tpr)))

plot_confusion_matrix(cnf_matrix, classes=labels,
                      title='Confusion matrix, without normalization')


plt.show()
t1 = time.time()
total = t1-t0

print(datetime.datetime.now())
print("total time deep NN and randomoversampler:{}" .format(total))

fpr, tpr, threshold = metrics.roc_curve(y_test, y_pred_1d)
roc_auc = metrics.auc(fpr, tpr)

# method I: plt
import matplotlib.pyplot as plt
plt.title('Receiver Operating Characteristic')
plt.plot(fpr, tpr, 'b', label = 'AUC = %0.2f' % roc_auc)
plt.legend(loc = 'lower right')
plt.plot([0, 1], [0, 1],'r--')
plt.xlim([0, 1])
plt.ylim([0, 1])
plt.ylabel('True Positive Rate')
plt.xlabel('False Positive Rate')
plt.show()
```

In [ ]:

```
from imblearn.over_sampling import SMOTE

print("Before OverSampling, counts of label '1': {}".format(sum(y_train==1)))
print("Before OverSampling, counts of label '0': {} \n".format(sum(y_train==0)))

sm = SMOTE(random_state=42, n_jobs=90)
X_train_res, y_train_res = sm.fit_sample(x_train, y_train)
t0 = time.time()

history=model.fit(X_train_res, y_train_res.ravel(),
                    batch_size=64,
                    epochs=50,
                    callbacks=callbacks_list,
                    validation_split=0.1)

y_softmax = model.predict(x_test)

y_test_1d = []
y_pred_1d = []

for i in range(0, len(y_softmax)):
    probs = y_softmax[i]
```

© 2020 Tiwari P et al. *JAMA Network Open.*

```
    predicted_index = np.argmax(probs)

    y_pred_1d.append(predicted_index)

labels=[0,1]

# Compute confusion matrix
cnf_matrix = confusion_matrix(y_test, y_pred_1d)
np.set_printoptions(precision=2)

from sklearn.metrics import f1_score
print("f-score metric in the testing dataset: {}%".format(f1_score(y_test, y_pre
d_1d, average='binary') ))

from sklearn import metrics
fpr, tpr, thresholds = metrics.roc_curve(y_test, y_pred_1d)
print("AUC of test dataset is: {}" .format(metrics.auc(fpr, tpr)))

plot_confusion_matrix(cnf_matrix, classes=labels,
                      title='Confusion matrix, without normalization')


plt.show()
t1 = time.time()
total = t1-t0

print(datetime.datetime.now())
print("total time deep NN and SMOTE:{}" .format(total))

fpr, tpr, threshold = metrics.roc_curve(y_test, y_pred_1d)
roc_auc = metrics.auc(fpr, tpr)

# method I: plt
import matplotlib.pyplot as plt
plt.title('Receiver Operating Characteristic')
plt.plot(fpr, tpr, 'b', label = 'AUC = %0.2f' % roc_auc)
plt.legend(loc = 'lower right')
plt.plot([0, 1], [0, 1],'r--')
plt.xlim([0, 1])
plt.ylim([0, 1])
plt.ylabel('True Positive Rate')
plt.xlabel('False Positive Rate')
plt.show()
```

In [ ]:

```
from imblearn.under_sampling import ClusterCentroids

print("Before OverSampling, counts of label '1': {}".format(sum(y_train==1)))
print("Before OverSampling, counts of label '0': {} \n".format(sum(y_train==0)))

sm = ClusterCentroids(random_state=42, n_jobs=90)
X_train_res, y_train_res = sm.fit_sample(x_train, y_train)
t0 = time.time()
```

© 2020 Tiwari P et al. *JAMA Network Open.*

```
history=model.fit(X_train_res, y_train_res.ravel(),
                    batch_size=64,
                    epochs=50,
                    callbacks=callbacks_list,
                    validation_split=0.1)

y_softmax = model.predict(x_test)

y_test_1d = []
y_pred_1d = []

for i in range(0, len(y_softmax)):
    probs = y_softmax[i]
    predicted_index = np.argmax(probs)
    y_pred_1d.append(predicted_index)

labels=[0,1]

# Compute confusion matrix
cnf_matrix = confusion_matrix(y_test, y_pred_1d)
np.set_printoptions(precision=2)

from sklearn.metrics import f1_score
print("f-score metric in the testing dataset: {}%".format(f1_score(y_test, y_pre
d_1d, average='binary') ))

from sklearn import metrics
fpr, tpr, thresholds = metrics.roc_curve(y_test, y_pred_1d)
print("AUC of test dataset is: {}" .format(metrics.auc(fpr, tpr)))

plot_confusion_matrix(cnf_matrix, classes=labels,
                      title='Confusion matrix, without normalization')


plt.show()
t1 = time.time()
total = t1-t0

print(datetime.datetime.now())
print("total time deep NN and ClusterCentroids:{}" .format(total))

fpr, tpr, threshold = metrics.roc_curve(y_test, y_pred_1d)
roc_auc = metrics.auc(fpr, tpr)

# method I: plt
import matplotlib.pyplot as plt
plt.title('Receiver Operating Characteristic')
plt.plot(fpr, tpr, 'b', label = 'AUC = %0.2f' % roc_auc)
plt.legend(loc = 'lower right')
plt.plot([0, 1], [0, 1],'r--')
plt.xlim([0, 1])
plt.ylim([0, 1])
```

© 2020 Tiwari P et al. *JAMA Network Open.*

```
plt.ylabel('True Positive Rate')

plt.xlabel('False Positive Rate')
plt.show()
```

In [ ]:

```
from imblearn.under_sampling import RandomUnderSampler


print("Before OverSampling, counts of label '1': {}".format(sum(y_train==1)))
print("Before OverSampling, counts of label '0': {} \n".format(sum(y_train==0)))

sm = RandomUnderSampler(random_state=42)
X_train_res, y_train_res = sm.fit_sample(x_train, y_train)
t0 = time.time()

history=model.fit(X_train_res, y_train_res.ravel(),
                    batch_size=64,
                    epochs=50,
                    callbacks=callbacks_list,
                    validation_split=0.1)

y_softmax = model.predict(x_test)

y_test_1d = []
y_pred_1d = []

for i in range(0, len(y_softmax)):
    probs = y_softmax[i]
    predicted_index = np.argmax(probs)
    y_pred_1d.append(predicted_index)

labels=[0,1]

# Compute confusion matrix
cnf_matrix = confusion_matrix(y_test, y_pred_1d)
np.set_printoptions(precision=2)

from sklearn.metrics import f1_score
print("f-score metric in the testing dataset: {}%".format(f1_score(y_test, y_pre
d_1d, average='binary') ))

from sklearn import metrics
fpr, tpr, thresholds = metrics.roc_curve(y_test, y_pred_1d)
print("AUC of test dataset is: {}" .format(metrics.auc(fpr, tpr)))

plot_confusion_matrix(cnf_matrix, classes=labels,
                      title='Confusion matrix, without normalization')


plt.show()
t1 = time.time()
total = t1-t0
```

© 2020 Tiwari P et al. *JAMA Network Open.*

```
print(datetime.datetime.now())
print("total time deep NN and RandomUnderSampler:{}" .format(total))

fpr, tpr, threshold = metrics.roc_curve(y_test, y_pred_1d)
roc_auc = metrics.auc(fpr, tpr)

# method I: plt
import matplotlib.pyplot as plt
plt.title('Receiver Operating Characteristic')
plt.plot(fpr, tpr, 'b', label = 'AUC = %0.2f' % roc_auc)
plt.legend(loc = 'lower right')
plt.plot([0, 1], [0, 1],'r--')
plt.xlim([0, 1])
plt.ylim([0, 1])
plt.ylabel('True Positive Rate')
plt.xlabel('False Positive Rate')
plt.show()
```

© 2020 Tiwari P et al. *JAMA Network Open.*

In [ ]:

```
!pip install keras
!pip install imblearn
```

In [3]:

```
#from MulticoreTSNE import MulticoreTSNE as TSNE
import pandas as pd
import glob
import numpy as np
import google.datalab.bigquery as bq
import pandas as pd
import tensorflow as tf
from tensorflow.contrib import lookup
from tensorflow.python.platform import gfile
import numpy
import keras as kr
from keras.preprocessing.text import Tokenizer
from keras.models import Sequential
from keras.layers import Dense, Activation, Dropout
from keras.layers.advanced_activations import LeakyReLU, PReLU
from keras.utils import multi_gpu_model
import itertools
from sklearn.metrics import confusion_matrix
import matplotlib.pyplot as plt
import datetime
import time
```

```
Using TensorFlow backend.
```

© 2020 Tiwari P et al. *JAMA Network Open.*

In [4]:

```
def plot_confusion_matrix(cm, classes,
                          normalize=False,
                          title='Confusion matrix',
                          cmap=plt.cm.Blues):
    """
    This function prints and plots the confusion matrix.
    Normalization can be applied by setting `normalize=True`.
    """
    plt.imshow(cm, interpolation='nearest', cmap=cmap)
    plt.title(title)
    plt.colorbar()
    tick_marks = np.arange(len(classes))
    plt.xticks(tick_marks, classes, rotation=0)
    plt.yticks(tick_marks, classes)

    if normalize:
        cm = cm.astype('float') / cm.sum(axis=1)[:, np.newaxis]
        #print("Normalized confusion matrix")
    else:
        1#print('Confusion matrix, without normalization')

    #print(cm)

    thresh = cm.max() / 2.
    for i, j in itertools.product(range(cm.shape[0]), range(cm.shape[1])):
        plt.text(j, i, cm[i, j],
                 horizontalalignment="center",
                 color="white" if cm[i, j] > thresh else "black")

    plt.tight_layout()
    plt.ylabel('True label')
    plt.xlabel('Predicted label')
```

In [4]:

```
%%bq query -n requests
SELECT
label,  age, sex, diag_med
 FROM `hdcmlmed.adhocs.v_afib`
```

In [ ]:

```
data = requests.execute(output_options=bq.QueryOutput.dataframe()).result()
from sklearn.utils import shuffle
data = shuffle(data)

from sklearn.preprocessing import StandardScaler
train_size = int(len(data) * .8)

train_icdmed = data['diag_med'][:train_size]
train_label = data['label'][:train_size]
```

© 2020 Tiwari P et al. *JAMA Network Open.*

```
train_label = data['label'][:train_size]

test_icdmed = data['diag_med'][train_size:]
test_label = data['label'][train_size:]

vocab_size = 200
tokenize = kr.preprocessing.text.Tokenizer(num_words=vocab_size,
                                           filters='!"#$%&()*+,-./:;<=>?@[\\]^_`
{|}~\t\n',
                                           lower=True,
                                           split=",",char_level=False)
tokenize.fit_on_texts(train_icdmed)
x_train = tokenize.texts_to_matrix(train_icdmed)
x_test = tokenize.texts_to_matrix(test_icdmed)

train_sex = data['sex'][:train_size]
test_sex = data['sex'][train_size:]
vocab_size = 2
tokenize = kr.preprocessing.text.Tokenizer(num_words=vocab_size,
                                           filters='!"#$%&()*+,-./:;<=>?@[\\]^_`
{|}~\t\n',
                                           lower=True,
                                           split=",",char_level=False)
tokenize.fit_on_texts(train_sex)
x_train_sex = tokenize.texts_to_matrix(train_sex)
x_test_sex = tokenize.texts_to_matrix(test_sex)

x_train=np.hstack((x_train,x_train_sex))
x_test=np.hstack((x_test,x_test_sex))

scaler = StandardScaler()
age=data['age']
age=scaler.fit_transform(age.reshape(-1,1))
age_train=age[:train_size]
age_test=age[train_size:]
x_train=np.hstack((x_train,age_train))
x_test=np.hstack((x_test,age_test))


from sklearn import preprocessing
encoder = preprocessing.LabelBinarizer()
encoder.fit(train_label)
y_train = encoder.transform(train_label)
y_test = encoder.transform(test_label)

from imblearn.over_sampling import SMOTE
from imblearn.combine import SMOTEENN
from imblearn.combine import SMOTETomek
from imblearn.over_sampling import RandomOverSampler
from imblearn.over_sampling import ADASYN
from sklearn import preprocessing
from imblearn.under_sampling import ClusterCentroids
from imblearn.under_sampling import RandomUnderSampler
```

© 2020 Tiwari P et al. *JAMA Network Open.*

```
print("Before OverSampling, counts of label '1': {}".format(sum(y_train==1)))
print("Before OverSampling, counts of label '0': {} \n".format(sum(y_train==0)))

sm = RandomOverSampler(random_state=42)
X_train_res, y_train_res = sm.fit_sample(x_train, y_train)
t0 = time.time()

from sklearn.ensemble import RandomForestClassifier
clf = RandomForestClassifier(n_estimators=20000, max_depth=5, n_jobs=90,random_s
tate=0)
clf.fit(X_train_res, y_train_res.ravel())

y_pre = clf.predict(x_test)

t1 = time.time()
total = t1-t0

cnf_matrix = confusion_matrix(y_test, y_pre)
from sklearn.metrics import f1_score
print("f-score metric in the testing dataset: {}%".format(f1_score(y_test, y_pre
, average='binary') ))
from sklearn import metrics
fpr, tpr, thresholds = metrics.roc_curve(y_test, y_pre)
print("AUC of test dataset is: {}" .format(metrics.auc(fpr, tpr)))
class_names = [0,1]
plt.figure()
plot_confusion_matrix(cnf_matrix , classes=class_names, title='Confusion matrix'
)
plt.show()
print(datetime.datetime.now())
print("total time all features RF and RandomOverSampling:{}" .format(total))

fpr, tpr, threshold = metrics.roc_curve(y_test, y_pre)
roc_auc = metrics.auc(fpr, tpr)

# method I: plt
import matplotlib.pyplot as plt
plt.title('Receiver Operating Characteristic')
plt.plot(fpr, tpr, 'b', label = 'AUC = %0.2f' % roc_auc)
plt.legend(loc = 'lower right')
plt.plot([0, 1], [0, 1],'r--')
plt.xlim([0, 1])
plt.ylim([0, 1])
plt.ylabel('True Positive Rate')
plt.xlabel('False Positive Rate')
plt.show()
```

© 2020 Tiwari P et al. *JAMA Network Open.*

In [ ]:

```
t0 = time.time()

from sklearn.naive_bayes import GaussianNB
clf = GaussianNB()
clf.fit(X_train_res, y_train_res.ravel())
y_pre = clf.predict(x_test)

t1 = time.time()
total = t1-t0

cnf_matrix = confusion_matrix(y_test, y_pre)
from sklearn.metrics import f1_score
print("f-score metric in the testing dataset: {}%".format(f1_score(y_test, y_pre
, average='binary') ))
from sklearn import metrics
fpr, tpr, thresholds = metrics.roc_curve(y_test, y_pre)
print("AUC of test dataset is: {}" .format(metrics.auc(fpr, tpr)))
class_names = [0,1]
plt.figure()
plot_confusion_matrix(cnf_matrix , classes=class_names, title='Confusion matrix'
)
plt.show()
print(datetime.datetime.now())
print("total time all features Naive Bayes and RandomOverSampler:{}" .format(tot
al))

fpr, tpr, threshold = metrics.roc_curve(y_test, y_pre)
roc_auc = metrics.auc(fpr, tpr)

# method I: plt
import matplotlib.pyplot as plt
plt.title('Receiver Operating Characteristic')
plt.plot(fpr, tpr, 'b', label = 'AUC = %0.2f' % roc_auc)
plt.legend(loc = 'lower right')
plt.plot([0, 1], [0, 1],'r--')
plt.xlim([0, 1])
plt.ylim([0, 1])
plt.ylabel('True Positive Rate')
plt.xlabel('False Positive Rate')
plt.show()
```

© 2020 Tiwari P et al. *JAMA Network Open.*

In [ ]:

```
from xgboost import XGBClassifier
import time
t0 = time.time()
gbm = XGBClassifier(max_depth=4, n_estimators=20000, learning_rate=0.05, nthread
=90).fit(X_train_res, y_train_res.ravel())
y_pre = gbm.predict(x_test)

t1 = time.time()
total = t1-t0

cnf_matrix = confusion_matrix(y_test, y_pre)
from sklearn.metrics import f1_score
print("f-score metric in the testing dataset: {}%".format(f1_score(y_test, y_pre
, average='binary') ))
from sklearn import metrics
fpr, tpr, thresholds = metrics.roc_curve(y_test, y_pre)
print("AUC of test dataset is: {}" .format(metrics.auc(fpr, tpr)))
class_names = [0,1]
plt.figure()
plot_confusion_matrix(cnf_matrix , classes=class_names, title='Confusion matrix'
)
plt.show()
print(datetime.datetime.now())
print("total time all features xgboost and RandomOverSampler:{}" .format(total))

fpr, tpr, threshold = metrics.roc_curve(y_test, y_pre)
roc_auc = metrics.auc(fpr, tpr)

# method I: plt
import matplotlib.pyplot as plt
plt.title('Receiver Operating Characteristic')
plt.plot(fpr, tpr, 'b', label = 'AUC = %0.2f' % roc_auc)
plt.legend(loc = 'lower right')
plt.plot([0, 1], [0, 1],'r--')
plt.xlim([0, 1])
plt.ylim([0, 1])
plt.ylabel('True Positive Rate')
plt.xlabel('False Positive Rate')
plt.show()
```

© 2020 Tiwari P et al. *JAMA Network Open.*

In [ ]:

```
t0 = time.time()
from sklearn.linear_model import LogisticRegression
clf = LogisticRegression(C=1000, class_weight=None, dual=False, fit_intercept=Tr
ue,
          intercept_scaling=1, max_iter=100, multi_class='ovr', n_jobs=60,
           random_state=None, solver='liblinear', tol=0.0001,
          verbose=0, warm_start=False)
clf.fit(X_train_res, y_train_res.ravel())
y_pre = clf.predict(x_test)
t1 = time.time()
total = t1-t0

cnf_matrix = confusion_matrix(y_test, y_pre)
from sklearn.metrics import f1_score
print("f-score metric in the testing dataset: {}%".format(f1_score(y_test, y_pre
, average='binary') ))
from sklearn import metrics
fpr, tpr, thresholds = metrics.roc_curve(y_test, y_pre)
print("AUC of test dataset is: {}" .format(metrics.auc(fpr, tpr)))
class_names = [0,1]
plt.figure()
plot_confusion_matrix(cnf_matrix , classes=class_names, title='Confusion matrix'
)
plt.show()
print(datetime.datetime.now())
print("total time l2 logistic regression and RandomOverSampler:{}" .format(total
))

fpr, tpr, threshold = metrics.roc_curve(y_test, y_pre)
roc_auc = metrics.auc(fpr, tpr)

# method I: plt
import matplotlib.pyplot as plt
plt.title('Receiver Operating Characteristic')
plt.plot(fpr, tpr, 'b', label = 'AUC = %0.2f' % roc_auc)
plt.legend(loc = 'lower right')
plt.plot([0, 1], [0, 1],'r--')
plt.xlim([0, 1])
plt.ylim([0, 1])
plt.ylabel('True Positive Rate')
plt.xlabel('False Positive Rate')
plt.show()
```

In [ ]:

```
from keras.models import Sequential
from keras.layers import Dense, Activation, Dropout
from keras.layers.advanced_activations import LeakyReLU, PReLU
from keras.utils import multi_gpu_model
from keras.applications import Xception
from keras.callbacks import EarlyStopping
```

© 2020 Tiwari P et al. *JAMA Network Open.*

```
from keras.callbacks import EarlyStopping

t0 = time.time()

earlystop = EarlyStopping(monitor='val_loss', min_delta=0.0001, patience=1, verb
ose=1, mode='auto')
callbacks_list = [earlystop]
model = Sequential()
model.add(Dense(100, input_shape=(x_train.shape[1],)))
model.add(Activation('tanh'))
model.add(Dropout(0.2))
model.add(Dense(100))
model.add(Activation('tanh'))
model.add(Dropout(0.2))
model.add(Dense(2, activation = 'softmax'))


adam = kr.optimizers.Adamax(lr=0.001, beta_1=0.9, beta_2=0.999, epsilon=1e-08, d
ecay=0.0)
model.compile(loss='sparse_categorical_crossentropy',
              optimizer=adam,
              metrics=['accuracy'])

history=model.fit(X_train_res, y_train_res.ravel(),
                    batch_size=64,
                    epochs=50,
                    callbacks=callbacks_list,
                    validation_split=0.1)

y_softmax = model.predict(x_test)

y_test_1d = []
y_pred_1d = []

for i in range(0, len(y_softmax)):
    probs = y_softmax[i]
    predicted_index = np.argmax(probs)
    y_pred_1d.append(predicted_index)

labels=[0,1]
def plot_confusion_matrix(cm, classes,
                          normalize=False,
                          title='Confusion matrix',
                          cmap=plt.cm.Blues):
    """
    This function prints and plots the confusion matrix.
    Normalization can be applied by setting `normalize=True`.
    """
    if normalize:
        cm = cm.astype('float') / cm.sum(axis=1)[:, np.newaxis]
        print("Normalized confusion matrix")
    else:
        print('Confusion matrix, without normalization')
```

© 2020 Tiwari P et al. *JAMA Network Open.*

```
    print(cm)


    plt.imshow(cm, interpolation='nearest', cmap=cmap)
    plt.title(title)
    plt.colorbar()
    tick_marks = np.arange(len(classes))
    plt.xticks(tick_marks, classes, rotation=45)
    plt.yticks(tick_marks, classes)

    fmt = '.2f' if normalize else 'd'
    thresh = cm.max() / 2.
    for i, j in itertools.product(range(cm.shape[0]), range(cm.shape[1])):
        plt.text(j, i, format(cm[i, j], fmt),
                 horizontalalignment="center",
                 color="white" if cm[i, j] > thresh else "black")

    plt.tight_layout()
    plt.ylabel('True label')
    plt.xlabel('Predicted label')

# Compute confusion matrix
cnf_matrix = confusion_matrix(y_test, y_pred_1d)
np.set_printoptions(precision=2)

from sklearn.metrics import f1_score
print("f-score metric in the testing dataset: {}%".format(f1_score(y_test, y_pre
d_1d, average='binary') ))

from sklearn import metrics
fpr, tpr, thresholds = metrics.roc_curve(y_test, y_pred_1d)
print("AUC of test dataset is: {}" .format(metrics.auc(fpr, tpr)))

plot_confusion_matrix(cnf_matrix, classes=labels,
                      title='Confusion matrix, without normalization')


plt.show()
t1 = time.time()
total = t1-t0

print(datetime.datetime.now())
print("total time shallow NN and randomoversampler:{}" .format(total))

fpr, tpr, threshold = metrics.roc_curve(y_test, y_pred_1d)
roc_auc = metrics.auc(fpr, tpr)

# method I: plt
import matplotlib.pyplot as plt
plt.title('Receiver Operating Characteristic')
plt.plot(fpr, tpr, 'b', label = 'AUC = %0.2f' % roc_auc)
plt.legend(loc = 'lower right')
plt.plot([0, 1], [0, 1],'r--')
plt.xlim([0, 1])
```

© 2020 Tiwari P et al. *JAMA Network Open.*

```
plt.ylim([0, 1])

plt.ylabel('True Positive Rate')
plt.xlabel('False Positive Rate')
plt.show()
```

In [ ]:

```
probs=[]
for i in range(0, len(y_softmax)):
    #probs = y_softmax[i]
    probs.append(y_softmax[i,1])

from sklearn.metrics import roc_curve, auc
y_scores = probs
fpr, tpr, thresholds =roc_curve(y_test, y_scores)
roc_auc = auc(fpr, tpr)

####################################
# The optimal cut off would be where tpr is high and fpr is low
# tpr - (1-fpr) is zero or near to zero is the optimal cut off point
####################################
i = np.arange(len(tpr)) # index for df
roc = pd.DataFrame({'fpr' : pd.Series(fpr, index=i),'tpr' : pd.Series(tpr, index
= i), '1-fpr' : pd.Series(1-fpr, index = i), 'tf' : pd.Series(tpr - (1-fpr), ind
ex = i), 'thresholds' : pd.Series(thresholds, index = i)})
roc.ix[(roc.tf-0).abs().argsort()[:1]]

# Plot tpr vs 1-fpr
fig, ax = plt.subplots()
plt.plot(roc['tpr'])
plt.plot(roc['1-fpr'], color = 'red')
plt.xlabel('1-False Positive Rate')
plt.ylabel('True Positive Rate')
plt.title('Receiver operating characteristic')
ax.set_xticklabels([])

from sklearn.metrics import roc_curve, auc,  precision_recall_curve
p, r, thresholds = precision_recall_curve(y_test, y_scores)
def adjusted_classes(y_scores, t):
    """
    This function adjusts class predictions based on the prediction threshold (t
).
    Will only work for binary classification problems.
    """
    return [1 if y >= t else 0 for y in y_scores]

def precision_recall_threshold(p, r, thresholds, t=0.5):
    """
    plots the precision recall curve and shows the current value for each
    by identifying the classifier's threshold (t).
    """

    # generate new class predictions based on the adjusted_classes
```

© 2020 Tiwari P et al. *JAMA Network Open.*

```
    # function above and view the resulting confusion matrix.

    y_pred_adj = adjusted_classes(y_scores, t)
    print(pd.DataFrame(confusion_matrix(y_test, y_pred_adj),
                       columns=['pred_neg', 'pred_pos'],
                       index=['neg', 'pos']))

    # plot the curve
    plt.figure(figsize=(8,8))
    plt.title("Precision and Recall curve ^ = current threshold")
    plt.step(r, p, color='b', alpha=0.2,
             where='post')
    plt.fill_between(r, p, step='post', alpha=0.2,
                     color='b')
    plt.ylim([0.5, 1.01]);
    plt.xlim([0.5, 1.01]);
    plt.xlabel('Recall');
    plt.ylabel('Precision');

    # plot the current threshold on the line
    close_default_clf = np.argmin(np.abs(thresholds - t))
    plt.plot(r[close_default_clf], p[close_default_clf], '^', c='k',
            markersize=15)

#precision_recall_threshold(p, r, thresholds, 0.5)

def plot_precision_recall_vs_threshold(precisions, recalls, thresholds):
    """
    Modified from:
    Hands-On Machine learning with Scikit-Learn
    and TensorFlow; p.89
    """
    plt.figure(figsize=(8, 8))
    plt.title("Precision and Recall Scores as a function of the decision thresho
ld")
    plt.plot(thresholds, precisions[:-1], "b--", label="Precision")
    plt.plot(thresholds, recalls[:-1], "g-", label="Recall")
    plt.ylabel("Score")
    plt.xlabel("Decision Threshold")
    plt.legend(loc='best')
plot_precision_recall_vs_threshold(p, r, thresholds)
```

In [ ]:

```
from sklearn import datasets
from sklearn.naive_bayes import GaussianNB
from sklearn.linear_model import LogisticRegression
from sklearn.ensemble import RandomForestClassifier
from sklearn.svm import LinearSVC
from sklearn.calibration import calibration_curve

train_samples = 100  # Samples used for training the models
```

© 2020 Tiwari P et al. *JAMA Network Open.*

```
dnn=model


# ##############################################################################
# Plot calibration plots

plt.figure(figsize=(10, 10))
ax1 = plt.subplot2grid((3, 1), (0, 0), rowspan=2)
ax2 = plt.subplot2grid((3, 1), (2, 0))

ax1.plot([0, 1], [0, 1], "k:", label="Perfectly calibrated")
for clf, name in [(dnn, 'deep neural network')]:
    model.fit(X_train_res, y_train_res.ravel(),
                    batch_size=64,
                    epochs=50,
                    callbacks=callbacks_list,
                    validation_split=0.1)
    probs=[]
    y_softmax = model.predict(x_test)
    for i in range(0, len(y_softmax)):
      probs.append(y_softmax[i,1])
    prob_pos = probs

    fraction_of_positives, mean_predicted_value = \
        calibration_curve(y_test, prob_pos, n_bins=10)

    ax1.plot(mean_predicted_value, fraction_of_positives, "s-",
             label="%s" % (name, ))

    ax2.hist(prob_pos, range=(0, 1), bins=10, label=name,
             histtype="step", lw=2)

ax1.set_ylabel("Fraction of positives")
ax1.set_ylim([-0.05, 1.05])
ax1.legend(loc="lower right")
ax1.set_title('Calibration plots  (reliability curve)')

ax2.set_xlabel("Mean predicted value")
ax2.set_ylabel("Count")
ax2.legend(loc="upper center", ncol=2)

plt.tight_layout()
plt.show()
```

© 2020 Tiwari P et al. *JAMA Network Open.*

```
import pandas as pd
import glob
import numpy as np
import google.datalab.bigquery as bq
import pandas as pd
import tensorflow as tf
from tensorflow.contrib import lookup
from tensorflow.python.platform import gfile
import numpy
import keras as kr
from keras.preprocessing.text import Tokenizer
from keras.models import Sequential
from keras.layers import Dense, Activation, Dropout
from keras.layers.advanced_activations import LeakyReLU, PReLU
from keras.utils import multi_gpu_model
import itertools
from sklearn.metrics import confusion_matrix
import matplotlib.pyplot as plt
import datetime
import time
```

```
/usr/local/envs/py3env/lib/python3.5/site-packages/h5py/__init__.py:36: FutureWarning: Conversion o
f the second argument of issubdtype from `float` to `np.floating` is deprecated. In future, it will be treat
ed as `np.float64 == np.dtype(float).type`.
  from ._conv import register_converters as _register_converters
Using TensorFlow backend.
```

```
%%bq query -n requests
select label, age, sex,
case when DxHighBloodPressure=1 then 1 else 0 end DxHighBloodPressure,
case when DxObesity=1 then 1 else 0 end DxObesity,
case when DxDiabetes=1 then 1 else 0 end DxDiabetes,
case when DxMIAD=1 then 1 else 0 end DxMIAD ,
case when DxHeartFailure=1 then 1 else 0 end DxHeartFailure,
case when DxVHD=1 then 1 else 0 end DxVHD
from `hdcmlmed.uchlds.afib_few_features`
```

© 2020 Tiwari P et al. *JAMA Network Open.*

```
data = requests.execute(output_options=bq.QueryOutput.dataframe()).result()
from sklearn.utils import shuffle
data = shuffle(data)

cols=  ['DxHighBloodPressure','DxObesity','DxDiabetes','DxMIAD','DxHeartFailure','DxVHD']
from sklearn.preprocessing import StandardScaler
train_size = int(len(data) * .8)

x_train= data[cols][:train_size]
train_label = data['label'][:train_size]

x_test = data[cols][train_size:]
test_label = data['label'][train_size:]

train_sex = data['sex'][:train_size]
test_sex = data['sex'][train_size:]
vocab_size = 2
tokenize = kr.preprocessing.text.Tokenizer(num_words=vocab_size,
                                  filters='!"#$%&()*+,-./:;<=>?@[\\]^_`{|}~\t\n',
                                  lower=True,
                                  split=",",char_level=False)
tokenize.fit_on_texts(train_sex)
x_train_sex = tokenize.texts_to_matrix(train_sex)
x_test_sex = tokenize.texts_to_matrix(test_sex)

x_train=np.hstack((x_train,x_train_sex))
x_test=np.hstack((x_test,x_test_sex))

scaler = StandardScaler()
age=data['age']
age=scaler.fit_transform(age.reshape(-1,1))
age_train=age[:train_size]
age_test=age[train_size:]
x_train=np.hstack((x_train,age_train))
x_test=np.hstack((x_test,age_test))


from sklearn import preprocessing
encoder = preprocessing.LabelBinarizer()
encoder.fit(train_label)
y_train = encoder.transform(train_label)
y_test = encoder.transform(test_label)

from imblearn.over_sampling import RandomOverSampler

print("Before OverSampling, counts of label '1': {}".format(sum(y_train==1)))
print("Before OverSampling, counts of label '0': {} \n".format(sum(y_train==0)))

sm = RandomOverSampler(random_state=42)
X_train_res, y_train_res = sm.fit_sample(x_train, y_train)
```

© 2020 Tiwari P et al. *JAMA Network Open.*

```
def plot_confusion_matrix(cm, classes,
                          normalize=False,
                          title='Confusion matrix',
                          cmap=plt.cm.Blues):
    """
    This function prints and plots the confusion matrix.
    Normalization can be applied by setting `normalize=True`.
    """
    plt.imshow(cm, interpolation='nearest', cmap=cmap)
    plt.title(title)
    plt.colorbar()
    tick_marks = np.arange(len(classes))
    plt.xticks(tick_marks, classes, rotation=0)
    plt.yticks(tick_marks, classes)

    if normalize:
        cm = cm.astype('float') / cm.sum(axis=1)[:, np.newaxis]
        #print("Normalized confusion matrix")
    else:
        1#print('Confusion matrix, without normalization')

    #print(cm)

    thresh = cm.max() / 2.
    for i, j in itertools.product(range(cm.shape[0]), range(cm.shape[1])):
        plt.text(j, i, cm[i, j],
                 horizontalalignment="center",
                 color="white" if cm[i, j] > thresh else "black")

    plt.tight_layout()
    plt.ylabel('True label')
    plt.xlabel('Predicted label')
```

© 2020 Tiwari P et al. *JAMA Network Open.*

```
t0 = time.time()
from sklearn.linear_model import LogisticRegression
clf = LogisticRegression(penalty='none',C=0, class_weight=None, dual=False, fit_intercept=True,
        intercept_scaling=1, max_iter=100, solver= 'newton-cg', multi_class='ovr', n_jobs=60,
         random_state=None, tol=0.0001,
        verbose=0, warm_start=False)
clf.fit(X_train_res, y_train_res.ravel())
y_pre = clf.predict(x_test)
t1 = time.time()
total = t1-t0

cnf_matrix = confusion_matrix(y_test, y_pre)
from sklearn.metrics import f1_score
print("f-score metric in the testing dataset: {}%".format(f1_score(y_test, y_pre, average='binary') ))
from sklearn import metrics
fpr, tpr, thresholds = metrics.roc_curve(y_test, y_pre)
print("AUC of test dataset is: {}" .format(metrics.auc(fpr, tpr)))
class_names = [0,1]
plt.figure()
plot_confusion_matrix(cnf_matrix , classes=class_names, title='Confusion matrix')
plt.show()
print(datetime.datetime.now())
print("total time logistic regression and RandomOverSampler:{}" .format(total))

fpr, tpr, threshold = metrics.roc_curve(y_test, y_pre)
roc_auc = metrics.auc(fpr, tpr)

# method I: plt
import matplotlib.pyplot as plt
plt.title('Receiver Operating Characteristic')
plt.plot(fpr, tpr, 'b', label = 'AUC = %0.2f' % roc_auc)
plt.legend(loc = 'lower right')
plt.plot([0, 1], [0, 1],'r--')
plt.xlim([0, 1])
plt.ylim([0, 1])
plt.ylabel('True Positive Rate')
plt.xlabel('False Positive Rate')
plt.show()
```

© 2020 Tiwari P et al. *JAMA Network Open.*

eMethods. Machine Learning Code Details

Hyperparameter Tuning

Grid search was used to identify the optimal hyperparameters for all models, as relevant to the available hyperparameters for each machine-learning approach, with range 3 – 9 hyperparameter values evaluated per search. For algorithms included in the *sklearn* package (see below), the *sklearn.model_selection.GridSearchCV* function was applied with 10-fold cross-validation. For neural networks, iterative random sampling of 10,000 records was performed using the *sklearn.utils.shuffle* package to generate a separate dataset for each value in the grid, and models were fit using each value within the grid. For *keras* package (i.e., neural networks), grid search was performed using different activation functions (sigmoid, tanh, LeakyReLU, PReLU), different numbers of neurons per layer (range 500 – 15000), different number of hidden layers (range 1 – 5), learning rate (range 0.001 – 0.05), dropout (range 0.2 – 0.5), and early stopping (with and without).

Resampling

For resampling comparison, we employed the *imblearn.over_sampling_RandomOverSampler, imblearn.over_sampling_SMOTE, imblearn.under_sampling.RandomUnderSampler, imblearn.under_sampling.ClusterCentroids, and imblearn.over_sampling_SMOTETomek* packages. Of note, SMOTE-Tomek did not achieve convergence after multiple hours of computation, and was excluded from this analysis.

Supervised Analysis

See above for hyperparameter search strategy for each model. L2 Regularized logistic regression was performed using *sklearn.linear_model.LogisticRegression* with inverse regularization strength (C parameter) of 1000, tolerance of 0.0001, and 'liblinear' optimization algorithm. Naïve Bayesian analysis was performed using *sklearn.naive_bayes* with default settings. Random forest classification was performed using *sklearn.ensemble.RandomForestClassifier* with 20000 estimators (trees), with max depth of 5, n_jobs of 90, and random state of 0. Shallow neural network was performed using *Keras* with a single fully connected layer of 100 neurons, with tanh activation and softmax output, with dropout of 20%, Adam optimization (Learning rate = 0.001, epsilon = $1x10^{-8}$, decay = 0.0) and sparse cross-entropy loss. Deep neural network included seven hidden layers with 100 neurons each, tanh activation, dropout 20%, Adam optimization (Learning rate = 0.005, epsilon = $1x10^{-8}$, decay = 0.0), and sparse cross-entropy loss and early stopping. Gradient boosted classifier was performed using *xgboost.XGBClassifier* and Random Forest classifier, using 20000 estimators, max depth of 4, learning rate of 0.05, and 90 threads.

Calibration curves

Calibration curves were created using *sklearn.calibration.calibration_curve* function with 10 bins for the final model, and three additional comparative models.

© 2020 Tiwari P et al. *JAMA Network Open.*

eTable 1. Top 30 Features Across Entire Population

OMOP ID	Name	Domain	Count
2108115	Collection of venous blood by venipuncture	Procedure	657139
2414397	Office or other outpatient visit for the evaluation and management of an established patient (expanded problem focused history; expanded problem focused examination; moderate complexity decision-making)	Procedure	564188
2414398	Office or other outpatient visit for the evaluation and management of an established patient (detailed history; detailed examination; moderate complexity decision-making)	Procedure	453841
2514435	Emergency department visit for the evaluation and management of a patient (expanded problem focused history; expanded problem focused examination; moderate complexity decision-making)	Procedure	381625
35605482	2 ML Ondansetron 2 MG/ML Injection	Drug	376787
2414392	Office or other outpatient visit for the evaluation and management of a new patient (detailed history; detailed examination; moderate complexity decision-making)	Procedure	376279
40220357	1000 ML Sodium Chloride 9 MG/ML Injection	Drug	373058
320128	Essential hypertension	Condition	363953
4214956	History of clinical finding in subject	Observation	352657
4132855	Immunization	Procedure	348731
19070869	Ondansetron 2 MG/ML Injectable Solution	Drug	325982
2514436	Emergency department visit for the evaluation and management of a patient (detailed history; detailed examination; moderate complexity decision-making)	Procedure	316464
40162515	Acetaminophen 325 MG / Hydrocodone Bitartrate 5 MG Oral Tablet	Drug	305538
4036803	General examination of patient	Procedure	299408
35603428	2 ML Fentanyl 0.05 MG/ML Injection	Drug	281951
2514437	Emergency department visit for the evaluation and management of a patient (comprehensive history; comprehensive examination; moderate complexity decision-making)	Procedure	279757

© 2020 Tiwari P et al. *JAMA Network Open.*

40231925	Acetaminophen 325 MG / Oxycodone Hydrochloride 5 MG Oral Tablet	Drug	278383
2314215	Therapeutic, prophylactic, or diagnostic injection (specify substance or drug); intravenous push, single or initial substance/drug	Procedure	277471
19135374	Calcium Chloride 0.0014 MEQ/ML / Potassium Chloride 0.004 MEQ/ML / Sodium Chloride 0.103 MEQ/ML / Sodium Lactate 0.028 MEQ/ML Injectable Solution	Drug	261544
2213418	Immunization administration (includes percutaneous, intradermal, subcutaneous, or intramuscular injections); 1 vaccine (single or combination vaccine/toxoid)	Procedure	256066
254761	Cough	Condition	243377
200219	Abdominal pain	Condition	230740
2414396	Office or other outpatient visit for the evaluation and management of an established patient (problem focused history; problem focused examination; straightforward medical decision making)	Procedure	230680
2314205	Intravenous infusion, hydration; each additional hour (List separately in addition to code for primary procedure)	Procedure	226050
40244026	Naloxone Hydrochloride 0.4 MG/ML Injectable Solution	Drug	218298
40232756	Oxycodone Hydrochloride 5 MG Oral Tablet	Drug	217914
2414393	Office or other outpatient visit for the evaluation and management of a new patient (comprehensive history; comprehensive examination; moderate complexity decision-making)	Procedure	215793
19005965	Ondansetron 4 MG Disintegrating Oral Tablet	Drug	214654
2314216	Therapeutic, prophylactic, or diagnostic injection (specify substance or drug); each additional sequential intravenous push of a new substance/drug (List separately in addition to code for primary procedure)	Procedure	212402
432867	Hyperlipidemia	Condition	212301

© 2020 Tiwari P et al. *JAMA Network Open.*

eTable 2. List of OMOP Concept Numbers for Features Used as Input

2108115	40232756	40240688	19020131	439926	138384	436962	437390
2414397	2414393	4144111	19075034	138525	35606552	197381	42872402
2414398	19005965	437663	40227012	40225811	19076953	43560451	35603429
2514435	2314216	27674	40756910	4175226	40232444	987250	43526851
35605482	432867	2414391	2213283	2314262	2314297	35605848	2514433
2414392	77670	433316	2211359	2314048	141693	435796	2314290
40220357	257011	46287618	2211327	444094	40163500	2211809	257012
320128	25297	42707627	35603636	19134047	35603432	201826	437833
4214956	378253	1551170	436070	2617177	19065567	442752	46271370
4132855	442077	4147961	4150062	40221387	19019419	35604998	42900442
19070869	46287424	19020053	4223659	19019074	2314047	40232700	444070
2514436	436096	42903088	1719046	19123989	134736	437264	40221384
40162515	194133	440383	2313869	439777	2314026	40180076	80180
4036803	2211361	19019073	2514434	2314217	938330	45768910	46275126
35603428	42902754	433736	40167259	75860	4237320	19041464	36717001
2514437	2212946	40180078	438485	2514399	442588	256451	19040121
40231925	2314213	19128020	140673	4169580	4306780	40240859	317009
2314215	46272450	31967	2314294	2314204	2314206	46257421	19080217
19135374	2212945	4014295	40232454	140214	44784779	1113346	260139
2213418	4167217	440029	19115197	778765	257007	2314287	40175249
254761	35603431	78232	1719012	2617289	441408	19078921	19044785
200219	46287338	24134	2514528	4215685	4209423	42708743	40223757
2414396	312437	81902	197684	437827	1836434	19023398	46276269
2314205	1127433	2314284	318800	19041324	40221386	438720	78508
40244026	196523	2212937	4193704	19078924	46287620	4088016	313459

© 2020 Tiwari P et al. *JAMA Network Open.*

eTable 3. Confusion Matrices

eTable 3A. Confusion Matrix of Best Model. AF = atrial fibrillation

		**Actual**	
**Predicted**		**AF**	**No AF**
	**AF**	4229 (0.94%)	67025 (14.88%)
	**No AF**	1394 (0.31%)	377790 (83.87%)

eTable 3B. Confusion matrix of known risk factors model based on logistic regression. AF = Atrial fibrillation

		**Actual**	
**Predicted**		**AF**	**No AF**
	**AF**	4682 (1.04%)	107506 (23.83%)
	**No AF**	995 (0.22%)	338040 (74.92%)

© 2020 Tiwari P et al. *JAMA Network Open.*

eTable 4. Coefficients for Logistic Regression Model

Risk Factor (ICD10, ICD9 code)	Beta coefficient
Hypertension (I10x, 401.x)	0.255
Obesity (E66.9, 278.0)	0.720
Diabetes Mellitus (E11.9, 250.00)	0.050
Coronary artery disease (I25.1, 414.01)	0.369
Heart failure (I50.9, 428.0)	0.799
Mitral Valve disease (I34.2, I34.0, 394.0, 424.0)	1.082
Female Sex	-0.357
Age (per SD)	1.430

Note: Coefficients are listed for logistic regression model with outcome 6-month incident atrial fibrillation. ICD = International Classification of Diseases; SD = standard deviation

© 2020 Tiwari P et al. *JAMA Network Open.*

eFigure. Calibration Curve of Final Model

Due to the low prevalence of AF across the UCHealth system (1.2%), the model was not well-calibrated, with most predicted probabilities being less than 20%.

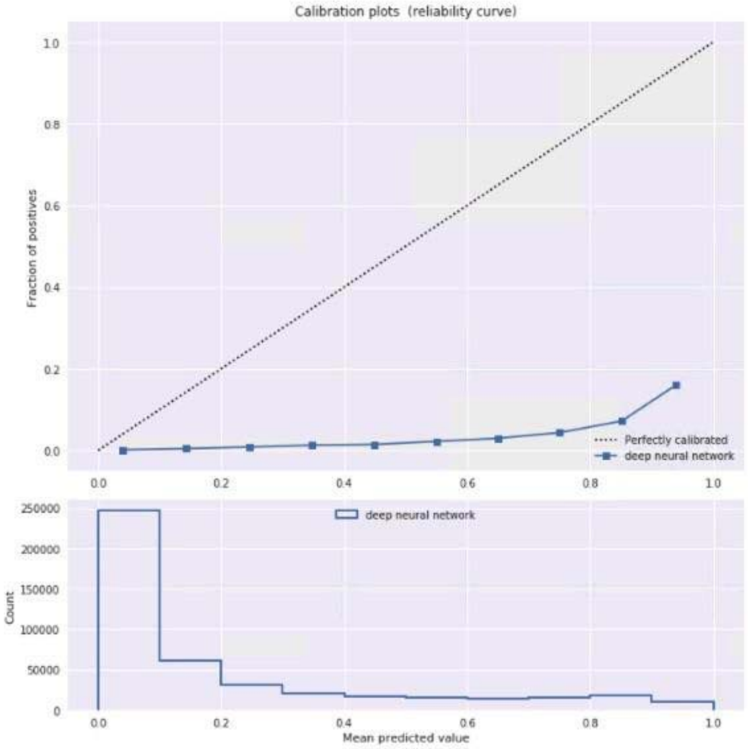

© 2020 Tiwari P et al. *JAMA Network Open.*
